# RIP1/RIP3/MLKL Mediates Myocardial Function Through Necroptosis in Experimental Autoimmune Myocarditis

**DOI:** 10.3389/fcvm.2021.696362

**Published:** 2021-08-23

**Authors:** Yujing Wu, Zhenzhong Zheng, Xiantong Cao, Qing Yang, Vikram Norton, Avner Adini, Amit K. Maiti, Irit Adini, Hao Wu

**Affiliations:** ^1^Department of Cardiology, The First Affiliated Hospital of Nanchang University, Nanchang, China; ^2^Department of Cardiology, Jiangxi Hypertension Research Institute, Nanchang, China; ^3^Department of Emergency, The First Affiliated Hospital of Xi'an Jiaotong University, Xi'an, China; ^4^Vascular Biology Program, Department of Surgery, Harvard Medical School, Boston Children's Hospital, Boston, MA, United States; ^5^Mydnavar, Department of Genetics and Genomics, Troy, MI, United States; ^6^Center for Engineering in Medicine, Harvard Medical School, Massachusetts General Hospital, Boston, MA, United States

**Keywords:** experimental autoimmune myocarditis, necroptosis, RIP1/RIP3/MLKL pathway, autophagy, necrostatin-1

## Abstract

Cardiomyopathy often leads to dilated cardiomyopathy (DCM) when caused by viral myocarditis. Apoptosis is long considered as the principal process of cell death in cardiomyocytes, but programmed necrosis or necroptosis is recently believed to play an important role in cardiomyocyte cell death. We investigated the role of necroptosis and its interdependency with other processes of cell death, autophagy, and apoptosis in a rat system of experimental autoimmune myocarditis (EAM). We successfully created a rat model system of EAM by injecting porcine cardiac myosin (PCM) and showed that in EAM, all three forms of cell death increase considerably, resulting in the deterioration of cardiac conditions with an increase in inflammatory infiltration in cardiomyocytes. To explore whether necroptosis occurs in EAM rats independent of autophagy, we treated EAM rats with a RIP1/RIP3/MLKL kinase-mediated necroptosis inhibitor, Necrostatin-1 (Nec-1). In Nec-1 treated rats, cell death proceeds through apoptosis but has no significant effect on autophagy. In contrast, autophagy inhibitor 3-Methyl Adenine (3-MA) increases necroptosis, implying that blockage of autophagy must be compensated through necroptosis. Caspase 8 inhibitor zVAD-fmk blocks apoptosis but increases both necroptosis and autophagy. However, all necroptosis, apoptosis, and autophagy inhibitors independently reduce inflammatory infiltration in cardiomyocytes and improve cardiac conditions. Since apoptosis or autophagy is involved in many important cellular aspects, instead of suppressing these two major cell death processes, Nec1 can be developed as a potential therapeutic target for inflammatory myocarditis.

## Introduction

Dilated cardiomyopathy (DCM) is a myocardial disease that is caused by viral infection or autoimmune or genetic factors. Some viral myocarditis (VMC) can develop into DCM, which leads to intractable congestive heart failure (CHF) and even sudden death (1). The development of VMC to DCM is mainly caused by progressive autoimmune injury. When cardiomyocytes are infected by pathogenic microorganisms, they generate an antigen epitope and trigger an autoimmune response (2).

Cell death is a fundamental process that has been classified as necroptosis, apoptosis, and autophagy (3). Over the years, necroptosis has been widely considered to be an unregulated, passive type of cell death that has been closely associated with many diseases. Its role in myocardial ischemia–reperfusion injury, cerebral ischemia, neuronal degeneration, immune diseases, tumors, and DCM has been established (4–7). It has long been known that apoptosis is vital to the maintenance of proper adaptive immune function. More recently, the contributions of additional cell death processes including cellular autophagy and necroptosis have been implicated in controlling both innate and adaptive immune functions (8).

Most studies involving cardiomyocyte cell death mainly focused on apoptosis. Necroptosis is a regulated, caspase-independent form of cell death, mediated by TNFR (tumor necrosis factor receptor), RIP1 (receptor interacting protein kinase 1), RIP3 (receptor interacting protein kinase 3), and MLKL (mixed lineage kinase domain-like). Accumulating evidence demonstrate that RIP1 and RIP3 form a RIP1–RIP3 complex (necrosome) that phosphorylates each other (9). MLKL oligomerizes and translocates to plasma membrane to form the RIP1/RIP3/MLKL complex to induce necroptosis (10). MLKL comprises an N-terminal helical bundle domain (HBD) and a C-terminal kinase-like domain of which HBD is crucial for the oligomerization of MLKL (11). The MLKL oligomerization results in ion channel dysfunction at the plasma membrane that causes an ion imbalance in both interior and exterior compartments of the membrane, which finally triggers necroptosis (12). When caspase-8 is inhibited with the apoptosis inhibitor zVAD-fmk, or suppressed with other agents, the RIP1 kinase combines with RIP3 to form necrosome. The kinase activity of RIP3, oligomerization of MLKL, and the necrosome formation are now considered to be crucial in the necroptosis signaling pathway (10, 13, 14).

Experimental autoimmune myocarditis (EAM) is an effective animal model to study the mechanism of myocardial injury. In the present study, we established an animal model of EAM in Lewis rats by immunizing a susceptible rat strain with porcine cardiac myosin (PCM) together with a strong adjuvant (15). The principle aim of this present study is to evaluate whether necroptosis is activated in EAM. Overall, our data show that the necroptotic pathway is triggered in EAM through the increased expression of RIP1, RIP3, and MLKL. As a result, the altered expression of these proteins deteriorates cardiac conditions and increases infiltration of inflammatory cells. Furthermore, we explored the possible effect of a small-molecule inhibitor of the necroptosis pathway, necrostatin-1 (Nec-1, a RIP1 kinase inhibitor), in myocardial cell injury and cardiac conditions in EAM (16). As expected, Nec-1 blocks RIP1/RIP3/MLKL-mediated necroptosis, reduces myocardial inflammatory infiltration, and improves cardiac conditions in EAM *in vivo*. Nec-1 does not show a significant effect on autophagy but shifts the response toward apoptosis. In contrast, suppression of autophagy by 3-MA increases necroptosis modulating proteins. Furthermore, apoptosis blockage by zVAD-fmk increases both necroptosis and autophagy. These results highlight the interrelation of necroptosis, apoptosis, and autophagy pathway genes in EAM and shed light on the pathogenic mechanism of myocarditis as well as indicate a novel therapeutic strategy for treatment of patients with myocarditis.

## Materials and Methods

### Animals and Experimental Study Design

Thirty-eight male Lewis rats, aged 8–9 weeks, weighing 200–220 g, specific-pathogen-free, were obtained from Vital River Laboratories (VRL), China, and all works were performed at Jiangxi Hypertension Research Institute, China. The study protocol was approved by the Institutional Review Board of the First Affiliated Hospital of Nanchang University. All experiments were conducted in accordance with the institutional guidelines for animal research and comply with the Guide for the Care and Use of Laboratory Animals published by the US National Institutes of Health (NIH publ. no. 85-23, revised 1996).

All rats are euthanized and sacrificed with the compliance of animal research ethical guidelines, and we strictly followed the instructions. Briefly, all rats in each group were administered, through subcutaneous injection, a single dose of 20% urethane (ethyl carbamate) solution (3 ml/kg of rat). Rats were then dissected, and their hearts were removed with incisions to the heart along with the coronal slitting. Half of the heart (longitudinal section keeping all four chambers) was fixed in 4% paraformaldehyde for histological examination; the other half of the heart was divided into two parts (again, longitudinally keeping all four chambers), one half is stored at −80°C for later Western Blotting tests, and the other half is saved in tissue preservation solution for RNA isolation.

**Figure 1:**
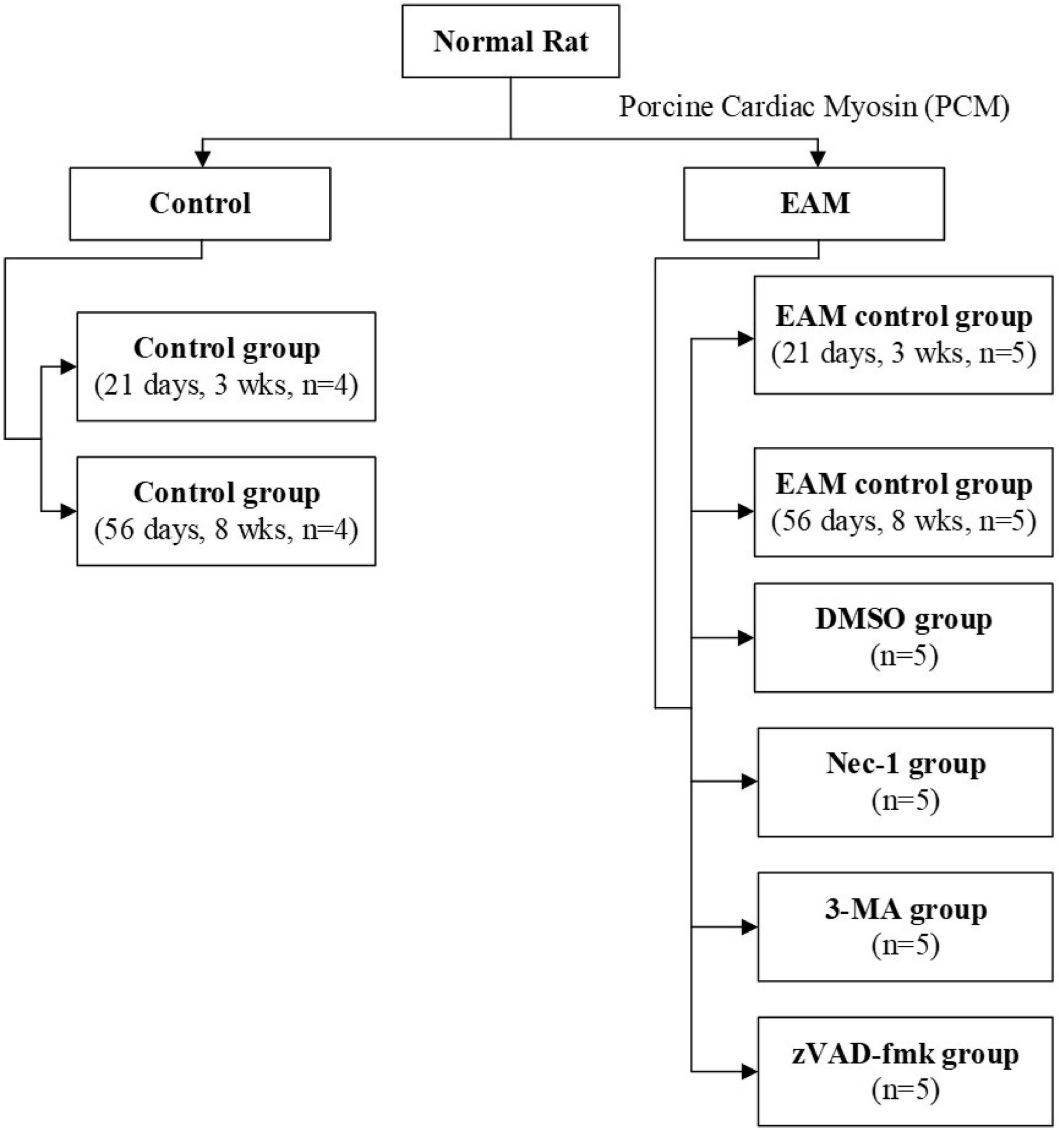
Flowchart of rats used in experiments.

Rats were kept under standard conditions with a mean temperature of 21 ± 2°C with a mean relative humidity of 50 ± 20% and a defined day-and-night rhythm of 12 h light and 12 h dark. Animals were randomized to control group 1 [21 days, 3 weeks (3W), *n* = 4], control group 2 (56 days, 8W, *n* = 4), and experimental group (*n* = 30). Experimental group rats were assigned (Figure 1) to five groups: the EAM group (21 days, 3W, *n* = 5; 56 days, 8W, *n* = 5), the necrostatin-1 group (Nec-1, *n* = 5), the zVAD-fmk group (zVAD-fmk, *n* = 5), the 3-Methyladenine group (3-MA, *n* = 5), and the DMSO group (*n* = 5). Rats of the control group (3W, *n* = 4), EAM group (3W, *n* = 5), Nec-1 group (*n* = 5), 3-MA group (*n* = 5), and DMSO group (*n* = 5) were sacrificed on 21 days (3W). Rats of the control group (8W, *n* = 4) and EAM group (8W, *n* = 5) were sacrificed on 56 days (8W) after the first injection.

### Inflammatory Myocarditis Induction

PCM (Sigma Aldrich, M0531) was diluted with 0.1 M PBS and finally emulsified with Complete Freund's Adjuvant (CFA, Sigma Aldrich, F5881) with a ratio of 1:1. Experimental group rats were subcutaneously injected with 0.2 ml of the PCM-CFA emulsion containing 1 mg of PCM in the footpad with a 7-day interval between injections (on days 0, 7, 14, and so on). Rats of the control group were subcutaneously injected with 0.1 ml of CFA alone on days 0 and 7. Control rats were sacrificed on 21 days (3W) and 56 days (8W) after the first injection.

### Drug Administration

Nec-1 (MedChem Express, HY-15760), zVAD-fmk (MedChem Express, HY-16658), and 3-MA (MedChem Express, HY-19312) were dissolved in 0.1% dimethylsulfoxide (DMSO, Sigma Aldrich, D2650) with 0.1 M phosphate-buffered saline (PBS). The drug dosages are: Nec-1 group, 0.6 mg/kg/day; zVAD-fmk group, 1.0 mg/kg/day; 3-MA group, 15 mg/kg/day; and DMSO group, 0.1% DMSO. Rats were injected intraperitoneally on day 0 and day 8, respectively. On day 21, rats were sacrificed after the first injection.

### Echocardiographic Examination

Transthoracic echocardiography was performed using a Sonos 5500 ultrasound machine (Phillips, USA.) with a 12-MHz phased array transducer with real-time digital acquisition, storage, and review capabilities. On day 21 and day 56, rats were anesthetized intraperitoneally with 3% chloral hydrate (0.01 ml/g) and the chests were shaved. Rats were placed on a shallow left lateral position. Two-dimensional mode, M-mode, and Doppler flow images were obtained in parasternal long/short-axis view. The left ventricular end-systolic and end-diastolic internal diameters (LVESd, LVEDd) were measured over the course of at least three consecutive cardiac cycles. The left ventricular ejection fraction (LVEF) and fractional shortening (FS) were calculated.

### Histopathological and Immunohistochemical Examination

The hearts were fixed in 4% paraformaldehyde in PBS and embedded in paraffin wax. Tissue sections (5 μm thick) were cut and stained with hematoxylin and eosin (H&E) to determine the level of inflammation. To evaluate the tissue-specific expression of RIP1, RIP3, MLKL, and LC3 I and II, we performed immunohistochemistry. In brief, control and the respective experimental group of tissue sections were incubated with mouse monoclonal anti-RIP1 antibodies (abcam, ab72139), rabbit polyclonal anti-RIP3 antibodies (abcam, ab62344), rabbit polyclonal anti-MLKL (Affinity Bioscience, DF7412), or rabbit polyclonal anti-LC3 I and II (Cell Signaling Technology, 12741S). The specificity of these antibodies was confirmed by Western blots (data not shown). The degree of myocardial infiltration and fibrosis was determined by two independent investigators in a double-blinded manner, and histology was scored as follows: 0, no infiltration; 1, <25%; 2, 25 to 50%; 3, 51 to 75%; 4, >75%.

#### Total RNA Extraction and RT-QPCR

All hearts were snap frozen and stored at −80°C. For the preparation of total RNA, the tissue was homogenized in Trizol reagent, extracted thrice with phenol-chloroform, and the total RNA was dissolved in 10 μl of RNase-free water. cDNA was prepared with PrimeScript™ RT reagent Kit with gDNA Eraser according to the kit specific protocol. Briefly, 10.0 μl of RNA, 2.0 μl of 5× gDNA Eraser Buffer, and 1.0 μl of gDNA Eraser were incubated at 42°C for 2 min. Reverse transcription was done with 1.0 μl of PrimeScript RT Enzyme Mix and 1.0 μl of RT Primer (random) Mix with 5 μl of 5× PrimeScript Buffer and 4.0 μl of 10 mM dNTP and made up the volume with RNase-free dH2O, incubated at 37°C for 1 h and then denatured at 85°C for 1 min. Real-time PCR was done with SYBR green method with SYBR Premix Taq. Briefly, 2× qPCR mix with SYBR Premix Taq 5 μl, primer (2.5 μm) 1 μl, cDNA 1 μl, ROX 0.2 μl, and dH2O (2.8 μl) with PCR cycle: 95°C, 1 min; 95°C, 40 s, 15 s → 58°C, and 20 s → 72°C. Dissolution curve was at 60°C → 95°C, with an increase of 1°C every 20 s. Forward and reverse primer sequences for each gene are given in Supplementary Table 1.

### Western Blotting Analysis

For Western analysis, total proteins were extracted using radio-immunoprecipitation assay (RIPA) lysis buffer (Solarbio, R0020). Protein was quantified by Varioskan (Thermo Scientific, USA) using a BCA protein assay kit (Beyotime, P0012S). Total protein was denatured by boiling for 10 min. Protein (40 μg) was separated by 10% SDS-PAGE and transferred to nitrocellulose filter membrane (Millipore, USA). After blocking with 5% non-fat milk/PBST for 1 h at room temperature, the membranes were incubated with the following primary antibodies in the respective experimental groups: mouse monoclonal anti-RIP1 antibodies (abcam, ab72139, 1:1,000 in 2% milk PBS), rabbit polyclonal anti-RIP3 antibodies (abcam, ab62344, 1:1,000 in 2% milk PBS), rabbit polyclonal anti-MLKL (Affinity Bioscience, DF7412, 1:1,000 in 2% milk PBS), and rabbit polyclonal anti-LC3 I and II (Cell Signaling Technology, 12741S, 1:1,000 in 2% milk PBS) for 12–16 h. Then, Peroxidase-Conjugated AffiniPure Goat anti-Rabbit IgG (H + L) (ZSGE-BIO, ZB-2301, 1:2,000 in 2% PBS) or Peroxidase-Conjugated AffiniPure Goat anti-Mouse IgG (H + L) (ZSGE-BIO, ZB-2305, 1:2,000 in 2% PBS) was added depending on the origin of primary antibodies for 2 h at room temperature and subsequently developed using an enhanced chemiluminescence reagent (Beyotime, P0018A). The signals emitted for chemiluminescence were detected using the ImageQuant LAS 4000 (Bio-Rad, 00746947) and analyzed with ImageJ software (NIH). The results were normalized to the corresponding densitometry signal of GAPDH (Abcam, 9484).

### Statistical Analysis

Data are presented as mean ± SEM. When comparing between two groups, data were statistically analyzed with a Student's two-tailed *t*-test. Differences among groups were determined by ANOVA followed by the Bonferroni's *post-hoc* test using SPSS version 20 (IBM SPSS, Armonk, NY) and GraphPad Prism 5 (GraphPad Software, Inc., CA, USA). *p*-values < 0.05 were considered statistically significant.

## Results

### RIP1/RIP3/MLKL-Mediated Necroptosis Induces Myocardial Injury in EAM Rat *in vivo*

**Figure 2:**
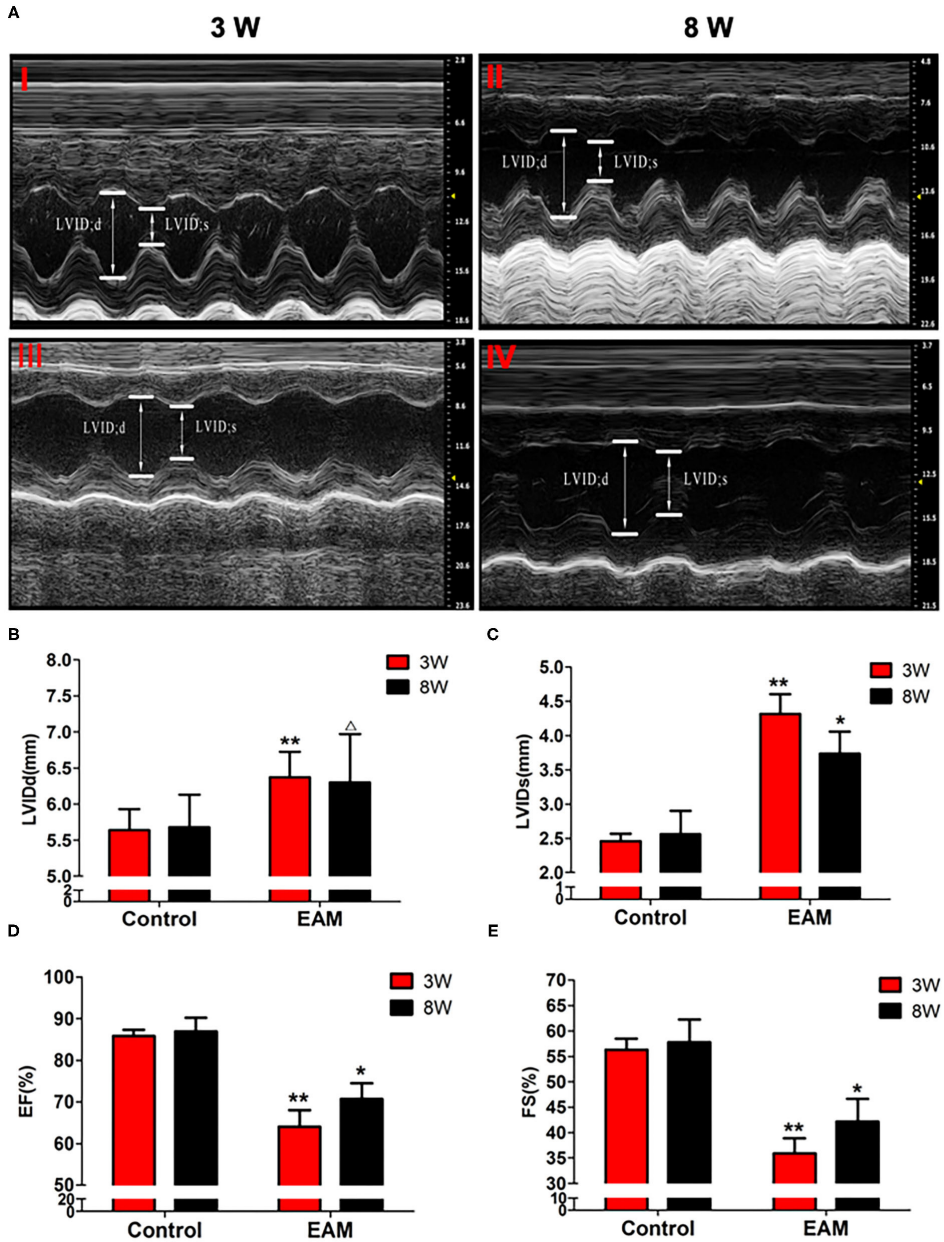
The level of LVIDd/LVIDs/EF/FS in “early” and “late” stages of myocarditis in control and EAM mice by echocardiography. **(A)** Control groups (top panel, 3W, I; 8W; II) and EAM mice (bottom panel, 3W,I II; 8W, IV). **(B–E)** Quantification of LVIDd **(B)**, LVIDs **(C)**, EF **(D)**, and FS **(E)** in “early” and “late” stages of myocarditis from control groups and EAM mice. *n* = 4 rat in control group, *n* = 5 rat per group **(B–E)**. All data are presented as mean ± SEM; a Student's *t*-test was performed to control group and EAM group. *p < 0.05 vs. control group; **p < 0.01 vs. EAM + DMSO group; ^Δ^*p* > 0.05 vs. EAM group.

Necroptosis is emerging as an important process of cell death in various cardiovascular pathological conditions. Although necroptosis influences the pathophysiological process during myocardial ischemia–reperfusion injury, acute myocardial infarction, acute heart failure, and atherosclerosis, no compelling evidence is reported for the association of necroptosis with EAM (17–19). To investigate whether necroptosis takes place in EAM, we designed experiments to understand the mechanisms of RIP1/RIP3/MLKL-mediated necroptosis and tested its effects in EAM *in vivo*. At 8–10 weeks of age of rats, there were no significant differences between the control group and experimental group in body weight and cardiac conditions (data not shown). PCM-treated rats during the phase of immunization on 3 and 8W had significant decreases in cardiac conditions and increases in myocardial inflammation compared to the control groups as revealed by echocardiographic examination of EF and FS (Figure 2) and immunohistological score of the myocarditis rats (Supplementary Figure 1). However, the EF values observed for control rats are higher than the value (64 ± 6.9) of Sprague–Dawley rats (20) but the Lewis rats considerably differ from Sprague–Dawley rats in heart size. EF measurements in Lewis rats are not reported elsewhere (21). Our measurement of EF value (85.152 ± 1.154%) may be relevant to our earlier report of EF value (81 ± 3.5%) in Lewis rats (22). However, deviation from 85.152 ± 1.154% to 63.9 ± 4.0% in EAM is significant, although it is within the normal range of the EF value of Sprague–Dawley rats.

Hearts from the EAM group of rats consistently showed dense inflammatory infiltrates on 3W and obvious myocardial fibrosis on 8W, whereas hearts from control group animals had only sparse lymphocytic infiltrations on both 3 and 8W. The deterioration of both cardiac conditions and myocardial inflammation in the experimental model over control group indicated that the EAM model was successfully established. Liu et al. (23) created a similar experimental model of Lewis rats with PCM that also showed similar inflammatory molecules in cardiac tissue with the increase of systemic serum inflammation with the increase of IFN*Γ*, IL2, IL4, and IL10 (23).

**Figure 3:**
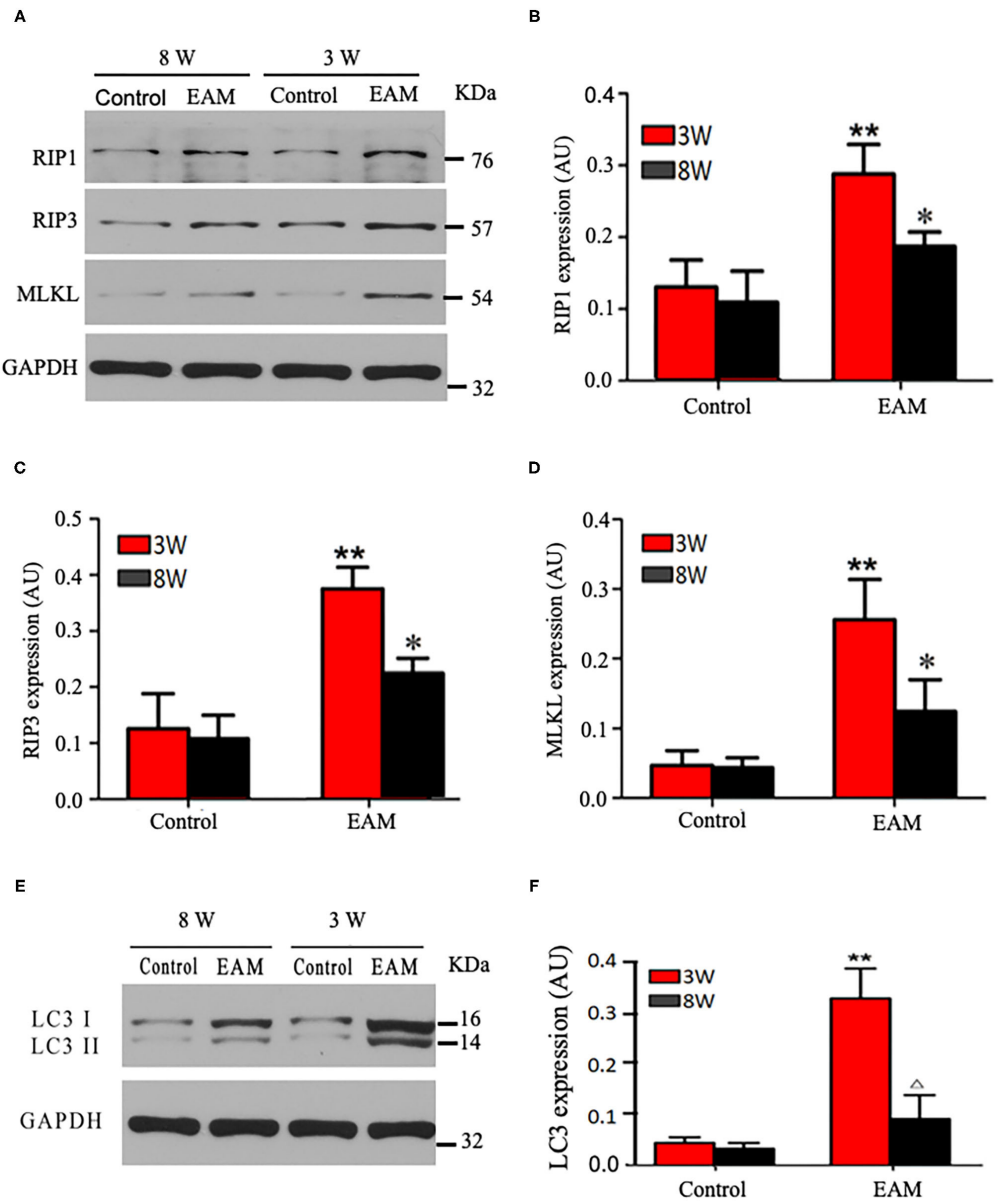
Protein expression levels of RIP1, RIP3, and MLKL in myocardial tissues of acute and chronic stages detected by Western blot. **(A)** Western blot analysis of protein expression of RIP1/RIP3/MLKL in rat myocardium during “early” and “late” stages. **(B–D)** Quantification of RIP1 **(B)**, RIP3 **(C)**, MLKL **(D)** in **(A)**. **(E)** Western blot analysis of marker protein expression of autophagosome LC-3 I and LC-3 II in rat myocardium during “early” and “late” stages. **(F)** Quantification of LC-3 I and LC-3 II in e. All data are presented as mean ± SEM. A Student's *t*-test was performed. **p < 0.05 means compared with control group (3W); *p < 0.05 means vs. control Group (8 weeks) comparison.

Next, we evaluated the potential effects of RIP1/RIP3/MLKL-induced necroptosis in myocardial injury in the EAM rat model. We investigated the expression of RIP1, RIP3, and MLKL in the early “stages” (day 21, 3W) and “late” stages (day 56, 8W) in EAM-induced myocardial injury. Notably, the mRNA and protein expression level of RIP1, RIP3, and MLKL were dramatically elevated in the EAM group compared to the control group on both 3 and 8W, as they were observed with Western blotting (Figures 3A–D) and RT-QPCR results for 3W (**Figures 5A–C**). Immunohistochemical examination also showed similar increases of RIP1, RIP3, and MLKL in the EAM group (**Figures 7A–D**). These results suggest that in both “early” and, “late” EAM rats, RIP1/RIP3-mediated necroptosis plays a critical role in developing myocardial injury and inflammatory infiltrates, although it is unclear whether necroptosis is the primary or principal pathway of cell death in EAM rats.

Next, we further tested the expression of CASPASE 3, BAX, BCL2, and LC3 I and II, for assessing apoptosis and autophagy, respectively, in the myocardium. CASPASE 3 is the primary executioner of the apoptosis pathway with increased BAX expression while increased BCL2 expression serves to protect cells from apoptosis. LC3 I and II, microtubule-associated proteins, are the accepted biological markers of autophagy pathway, and their expression in myocardium should indicate autophagosome formation, reflecting the extent of autophagy. The increase of CASPASE 3 and BAX and the decrease of BCL2 [Supplementary Figure 3; **Figures 5D–F** (EAM group, 3W)] in myocardial cells indicate an increase in apoptosis in EAM rats. Also, the increased protein expression of LC3 I and II (Figures 3E,F; Supplementary Figure 2) in both “early” and “late” stages of EAM rat myocardial tissue are observed in comparison to control groups on 3 and 8W. Taken together, these data confirm that in EAM rats, all three types of programmed cell death, necroptosis, apoptosis, and autophagy occur *in vivo*. In addition, the EAM group in “early” stages (3W) developed severe cardiac dysfunction (Figure 2), including decrease of EF and FS, ventricular dilation, and hypertrophy with reduced RIP1, RIP3, and MLKL expression. Thus, this group of rats (3W) could serve as an excellent EAM model for future use.

### Necroptosis Inhibitor Necrostatin (Nec-1) Prevents Necroptosis Without Affecting Autophagy in EAM *in vivo*

**Figure 4:**
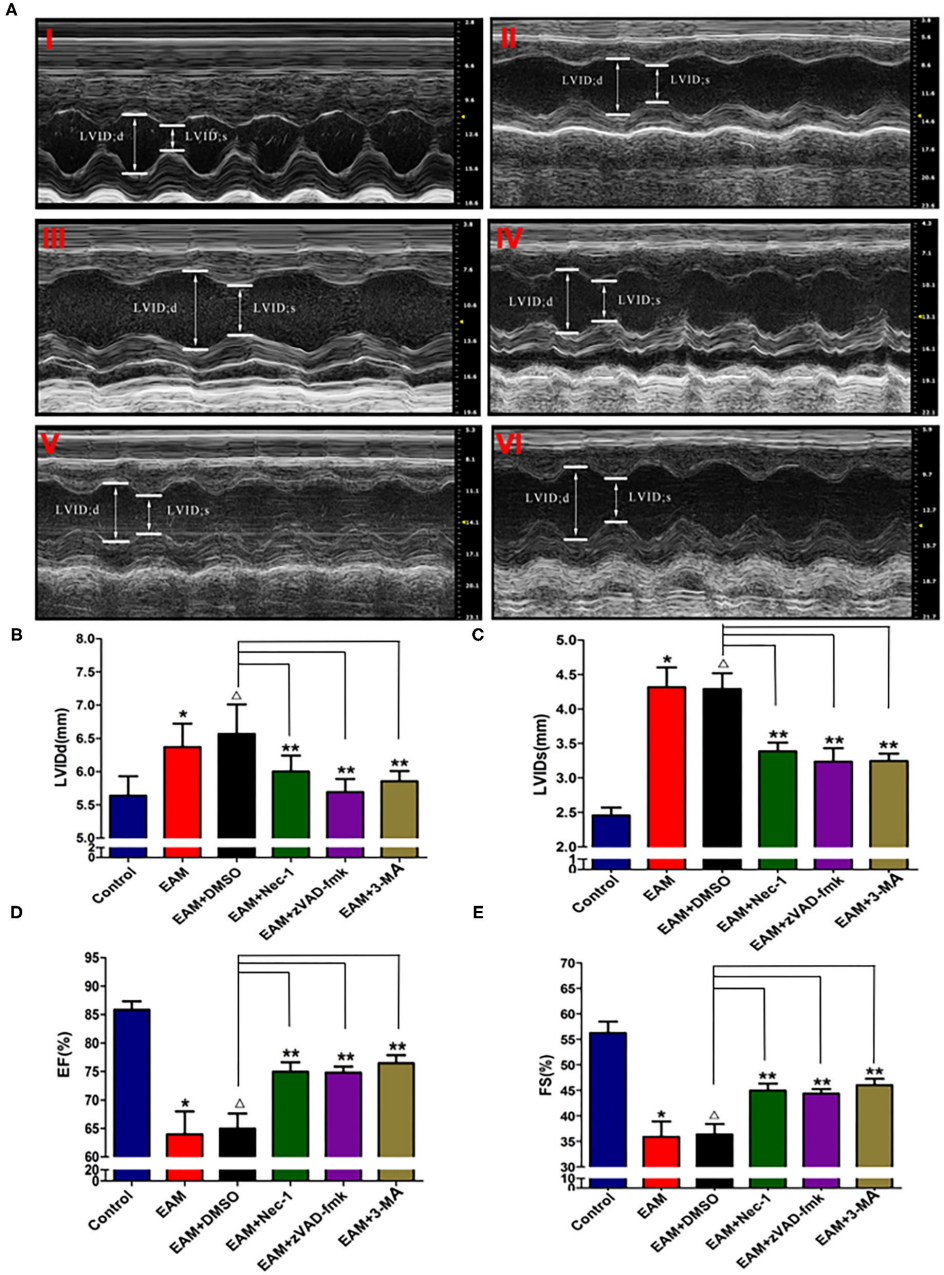
The level of LVIDd/LVIDs/EF/FS in “early” stage of myocarditis in EAM mice with inhibitors by echocardiography. **(A)** Echocardiographic images of control and EAM mice (3W) (I—control, II—EAM, III—EAM + DMSO, IV—EAM + Nec-1, V—EAM + zVAD-fmk, VI—EAM+3Ma). **(B–E)** Quantification of LVIDd, LVIDs, EF, and FS in the “early” stage of myocarditis from control groups. A horizontal bar is shown to indicate compared groups. All data are presented as mean ± SEM. A Student's *t*-test was performed for the control group and the EAM group, *p < 0.05 vs. control group **p < 0.01 vs. EAM + DMSO group, ^Δ^*p* > 0.05 vs. EAM group.

**Figure 5:**
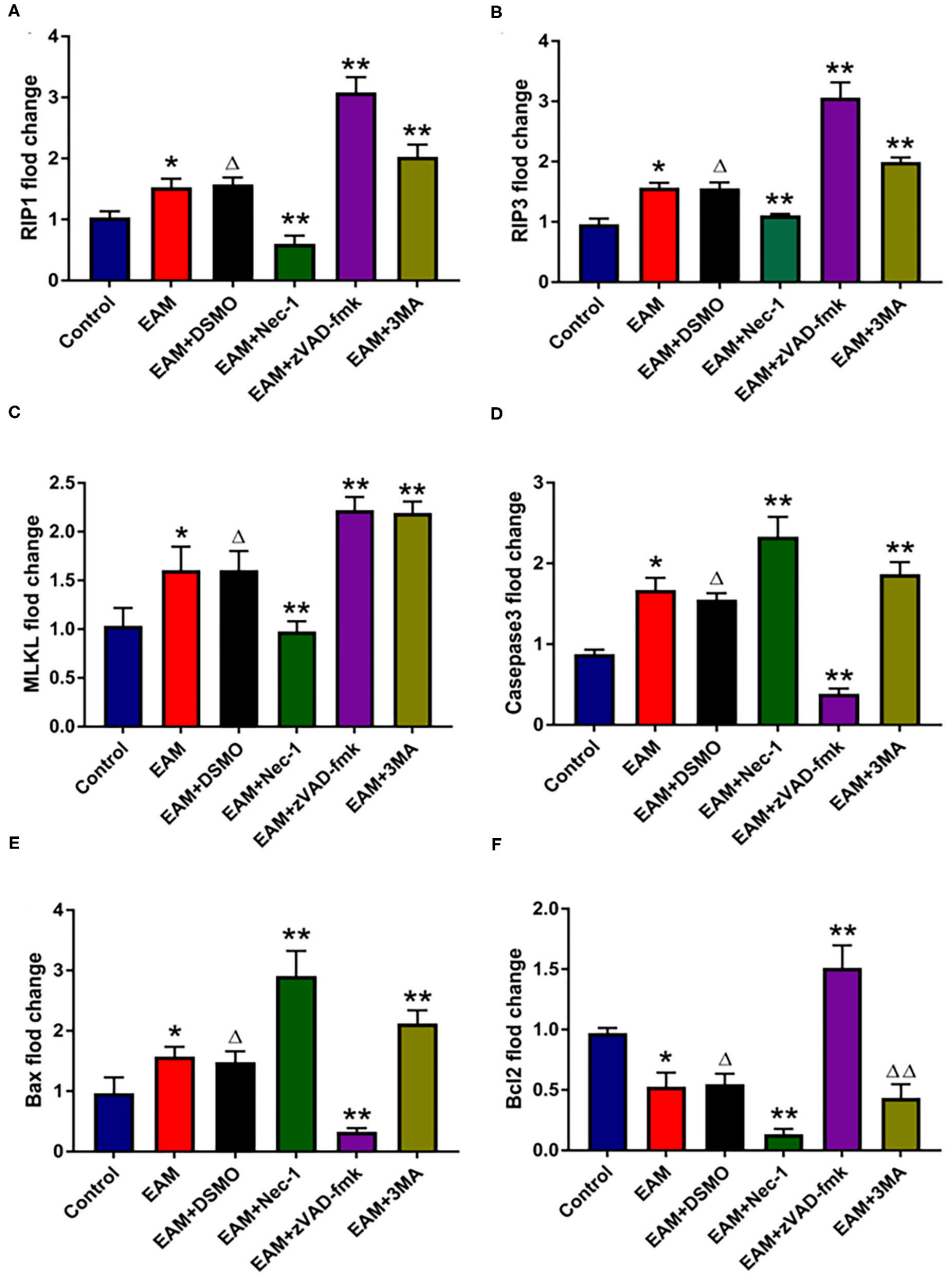
The relative expression of mRNA in myocardial tissue of each group detected by quantitative real-time PCR. **(A–C)** Real-time PCR analysis of RIP1, RIP3, and MLKL in each group, and inhibited by necrosis inhibitor Nec-1, and 3-MA but increased by zVAD-fmk. For EAM, 3W rats are used. **(D–F)** Real-time PCR analysis of Caspase 3, Bax, and Bcl2 in each group. The necrosis inhibitor Nec-1 and 3-MA increase the mRNA expression levels of Caspase 3/Bax mRNA in myocardial tissues. *p < 0.05 vs. control group; **p < 0.01 vs. EAM + DMSO group, ^**Δ**^*p* > 0.05 vs. EAM group, ^**ΔΔ**^*p* > 0.05 vs. EAM + DMSO group. A Student's *t*-test was performed for the control group and the EAM group, one-way analysis of variance (ANOVA) followed by the LSD test; data are presented as mean ± SEM.

**Figure 6:**
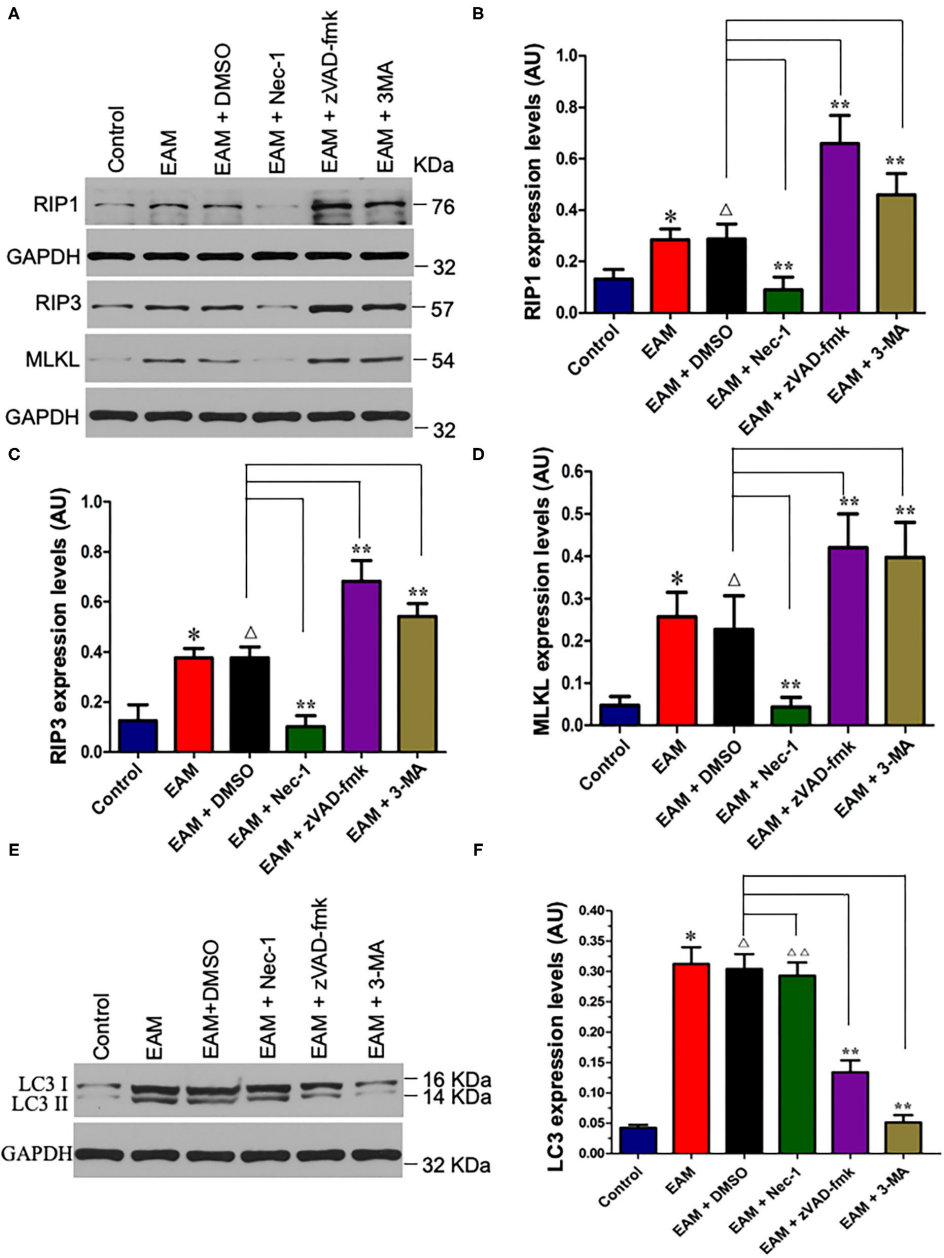
Nec-I inhibits but 3-MA increases the expression levels of RIP1, RIP3, and MLKL in EAM. **(A)** Western analysis of protein expression levels of RIP1, RIP3, and MLKL in myocardial tissues from the control group; EAM (3W) group; EAM + DMSO group; and EAM + Nec-I, EAM + zVAD-fmk, and EAM + 3MA drug group. **(B–D)** Quantification of protein levels of RIP1 in b, RIP3 in c, and MLKL in d. Horizontal lines above bars indicate groups compared. All data are presented as mean ± SEM. A Student's *t*-test was performed for the control group and EAM group, one-way analysis of variance (ANOVA) followed by the LSD test. *p < 0.05 vs. control group; **p < 0.01 vs. EAM + DMSO group, ^Δ^*p* > 0.05 vs. EAM group. ^Δ^*p* > 0.05 vs. EAM + Nec-1 group, ^Δ^*p* > 0.05 vs. EAM + 3-MA group. **(E,F)** Quantification of LC-3 I and II. Western analysis of LC-3 I and II expression levels treated by inhibitors of Nec-1 and 3-MA. **(D)** Quantification of LC-3 I and II of EAM (3W); ^ΔΔ^*p* < 0.05 means compared with the control group (3 weeks); **p > 0.05 means compared with the control group (8 weeks).

**Figure 7:**
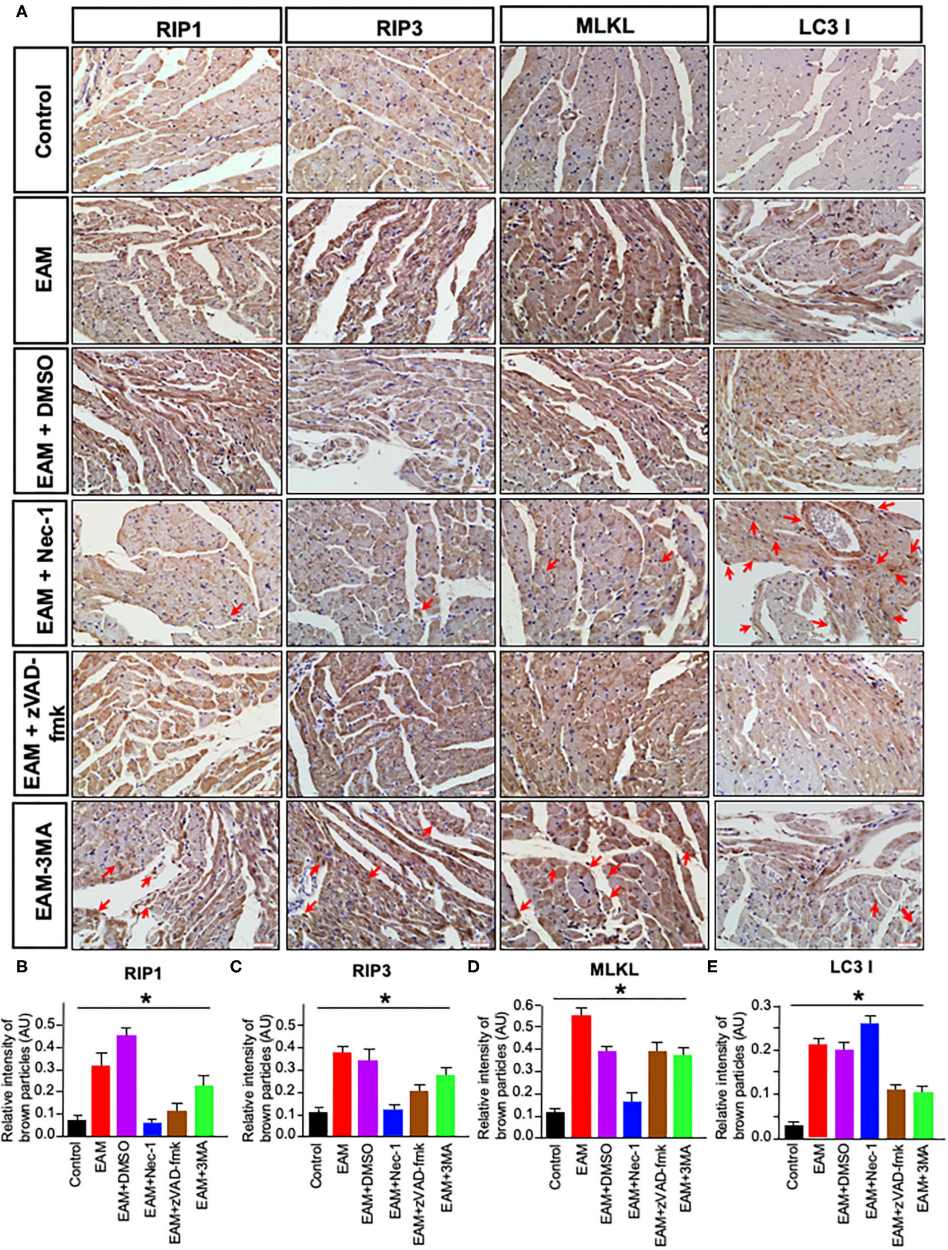
Immunohistochemical staining of RIP1/RIP3/MLKL and LC3B in rat myocardium. **(A)** Imunohistochemistry staining images of RIP1/RIP3/MLKL and LC3B. Red arrows point the brown particle deposition. Control: control group; EAM: EAM (3W); IMM + DMSO; EAM + Nec-1: necroptosis inhibitor group; EAM-zVAD-fmk, apoptosis inhibitor group, EAM + 3-MA: autophagy inhibitor group. RIP1, RIP3, MLKL, and LC3B. **(B–E)** Quantity of brown–red particles' intensity in the cytoplasm of RIP1, RIP3, MLKL, and LC3 I, respectively. The necrosis inhibitor Nec-1 decreased the intensities in RIP1, RIP3, and MLKL, but not in LC3 I and II. However, the autophagy inhibitor 3-MA inhibited the intensity of LC3B. A Student's *t*-test was performed in each group. *p < 0.05 are obtained for RIP1, when comparing EAM with EAM + Nec-1, EAM + zVAD-fmk, 3-MA; for RIP3, EAM with EAM + Nec-1, EAM + zVAD-fmk, and EAM + 3-MA; for MLKL, EAM with EAM-Nec-1, EAM + zVAD-fmk, and EAM-3-MA; for LC3B, EAM and EAM + Nec-1 and EAM and EAM +3-MA.

**Figure 8:**
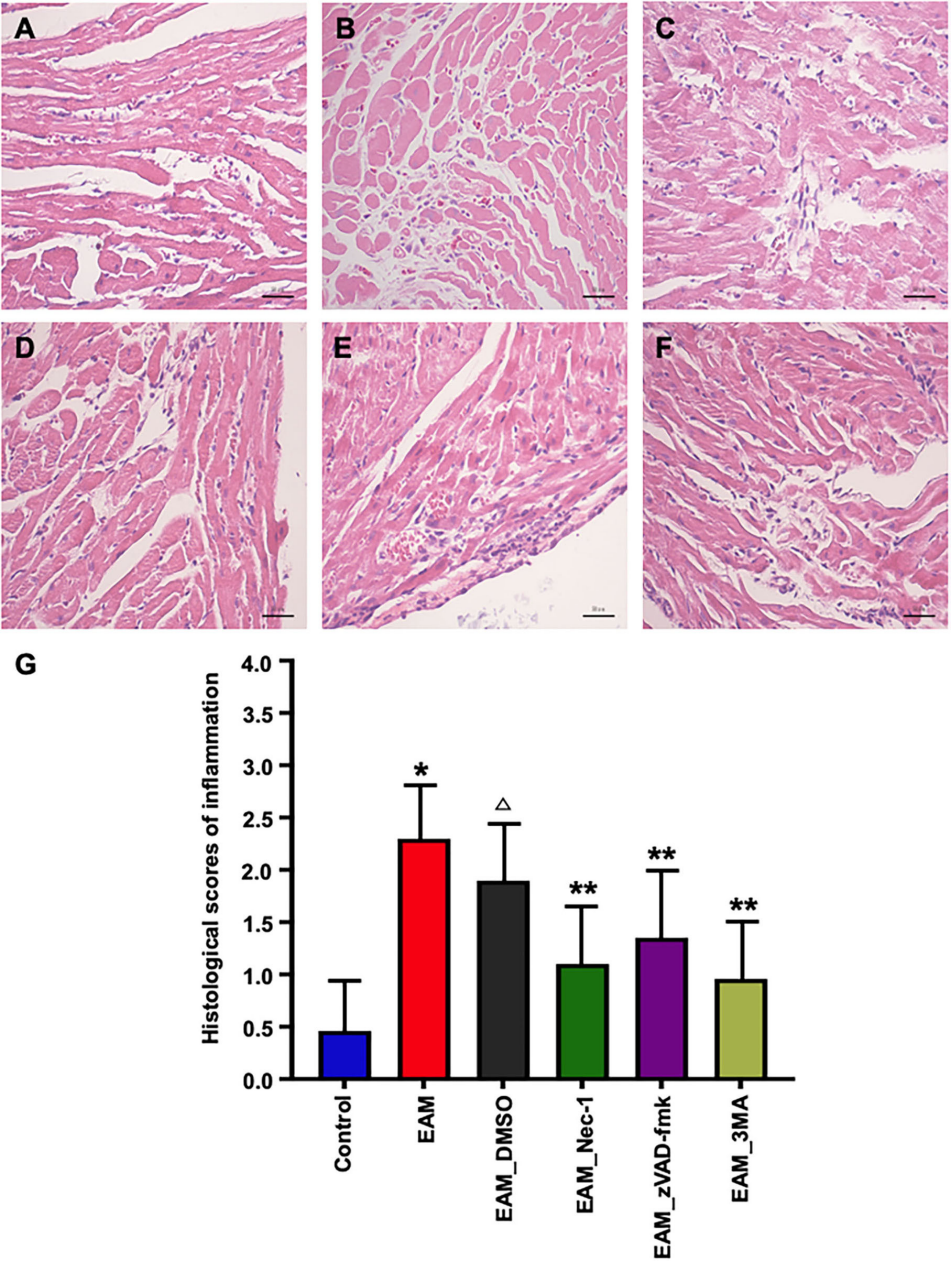
Pathological evaluations of myocardial tissues of various groups by HE staining. **(A)** In the heart of control group rat, there was no inflammatory infiltrate. **(B,C)** HE staining showed dense inflammatory infiltrates in the EAM **(B)** group (3W) and IMM + DMSO group **(C)**. Treating rat with Nec-1, zVAD-fmk, and 3-MA on day 21 attenuated inflammatory infiltrates. Only little scattered inflammatory infiltrates were detected in the EAM + Nec-1 group **(D)**. **(G)** Quantification of cellular inflammatory infiltration in **(A–F)**. All data are presented as mean ± SEM. A Student's *t*-test was performed for the control group and IMM group, one-way analysis of variance (ANOVA) followed by the LSD test. *p < 0.05 vs. control group, **p < 0.01 vs. EAM + DMSO group, *p* < 0.05 vs. EAM. ^Δ^ Compared with EAM group, the EAM_DMSO group has no significance.

Nec-1 is a small molecule specific inhibitor of necroptosis that uniquely inhibits the activation of RIP1 and the interaction of RIP1 with RIP3 to form the RIP1–RIP3 complex to subsequently block necroptosis (24, 25). To understand whether Nec-1 inhibits RIP1/RIP3/MLKL-dependent necroptosis in EAM rats *in vivo* and whether this inhibition is independent of autophagy, we treated “early” stages of EAM group (3W) rats with necrostatin injection (Nec-1 group). Strikingly, we observed that in the Nec-1 group, cardiac conditions were improved, inflammatory infiltration was ameliorated, myocardial cell injury was attenuated, and, thus, most of the pathological lesions of DCM in Nec-1 group were alleviated compared to the EAM-DMSO group (Figures 4, **8A–F**). We assessed the expression of RIP1, RIP3, MLKL, CASPASE 3, BAX, BCL2, and LC3 I and II in Nec-1-treated rats. The results of RT-QPCR, Western blotting, and immunohistochemical studies revealed that the expression levels of RIP1, RIP3, and MLKL were significantly decreased (Figures 5A–C, 6A–D, 7A–D) in the Nec-1 group compared to the EAM + DMSO group and there were no differences in the expression levels of these genes and proteins (RIP1, RIP3, and MLKL) between the non-treated EAM and DMSO groups. Meanwhile, we also observed that treatment with Nec-1 increased the mRNA expression of CASPASE 3 and BAX and decreased BCL2 expression (Figures 5D–F). Notably, it did not obviously affect the expression level of LC3 I and II in the Nec-1 group compared to the EAM-DMSO group (Figures 6E,F). However, the expression levels of these two genes and proteins were also unchanged in the non-treated EAM and DMSO groups. These results demonstrate that administration of Nec-1 in the “early” stages of EAM inhibits RIP1/RIP3/MLKL-dependent necroptosis, increases apoptosis, but has no effect on the autophagy pathway in the EAM Nec-1 group *in vivo*. Nevertheless, Nec-1 significantly preserved cardiac function and reduced inflammatory infiltrates in the EAM model, highlighting the importance of its cardioprotective effects, suggesting that it could be developed as future therapeutic and diagnostic targets for inflammatory myocarditis.

### Autophagy Inhibitor 3-Methyl Adenine Triggers Cardiac Necroptosis and Apoptosis in EAM Rats *in vivo*

Autophagy, a caspase-independent pathway, is physiologically a cellular mechanism for cell growth regulation with internal homeostasis. Recent studies show that autophagy also occurs during necrosis (26). Nevertheless, it remains a conjecture whether 3-MA can shift cell death from autophagy toward RIP1/RIP3/MLKL-mediated necroptosis. To test this, we treated the “early” stages of EAM rats with the autophagy inhibitor 3-MA (3-MA group) and then assessed the expression of RIP1, RIP3, MLKL, and LC3 I and II. Our results demonstrated that LC3 I and II levels were decreased (Figures 6E,F), whereas RIP1, RIP3, and MLKL levels were elevated in the 3-MA rat groups compared to the EAM-DMSO group (Figures 6A–D). Immunohistochemical studies for these proteins also showed similar patterns as expression differences (Figure 7). These results indicate that the blockade of autophagy by 3-MA can induce the RIP1/RIP3/MLKL-mediated necroptosis pathway in EAM rats in “early” stages. Autophagy blockade by 3-MA also induces apoptosis as observed by the increased expression of Caspase3 and BAX.

Next, we also observed that cardiac conditions (measured by EF and FS) in the 3-MA group were significantly increased compared to the EAM-DMSO group as shown in the echocardiographic examination on 3W (Figure 4). In addition, HE staining showed that in the 3-MA group, inflammatory infiltration was reduced, which alleviated myocardial cells compared to the DMSO group (Figure 8). These results demonstrate that treatment with 3-MA also significantly preserves cardiac function and reduces inflammatory response in EAM.

### Blockade of Apoptosis by zVAD-Fmk Triggers Necroptosis and Autophagy in EAM Rats *in vivo*

The inhibitor of apoptosis, such as zVAD-fmk, influences the RIP3-induced programmed necroptosis (9, 14). To observe the influence of apoptosis on necroptosis and autophagy, we use zVAD-fmk, a CASPASE 8 inhibitor of apoptosis, to block the caspase-dependent apoptosis pathway and then monitored the expression level of necroptosis inducing genes, RIP1, RIP3, MLKL, apoptosis specific CASPASE 3, and autophagy specific LC3 I and II. We observed that inhibition of caspase-dependent apoptosis by zVAD-fmk dramatically enhanced necroptosis and autophagy, as assessed by increased expression of RIP1, RIP3, MLKL (Figures 6A–D, 7), and LC3 I and II (Figures 6E,F). Furthermore, EAM rats in the zVAD-fmk group had improved cardiac conditions (Figure 4) with reduced inflammatory infiltration and attenuated myocardial cell injury in comparison to the EAM-DMSO group (Figure 8). These results demonstrate that an apoptosis inhibitor, similar to a necroptosis inhibitor and an autophagy inhibitor, also significantly improves cardiac conditions and reduces inflammatory response in EAM rats. Thus, inhibition of the apoptosis pathway not only shifts myocardial cell death from apoptosis to necroptosis but also triggers autophagy and all the three major types of programmed cell death in EAM rats.

## Discussion

Myocarditis is an inflammatory disease and is one of the leading causes of DCM. DCM is the leading global event for heart transplantation that has an estimated prevalence of ~1:250 people (27). DCM can be detected in patients with progressive heart failure, arrhythmias, thromboembolic disease, or sudden cardiac death (SCD). It is characterized by left ventricular cavity enlargement or dilatation and systolic dysfunction (28). DCM is implicated as the main cause of adult death and can be induced by viral infection and autoimmune and genetic factors (27, 29). Current studies demonstrate that autoimmunity-mediated cardiac damage plays an important role in the pathogenesis of a subset of post-infectious myocarditis, progressive inflammation, fibrosis, and pathological remodeling in DCM (30).

EAM is an effective animal model to study the mechanism of myocardial injury. EAM can be induced in susceptible animals, such as balb/c mouse and Lewis rats, by immunization with PCM or with specific peptides or adoptive transfer of myosin-reactive T cells together with Freud's adjuvant (31). In EAM, CD4+ T cells induce the development of myocarditis, which is characterized by mononuclear macrophage infiltration, necrosis of cardiomyocytes, and proliferation of cardiac fibroblasts (32). To investigate the role of necroptosis, apoptosis, and autophagy, we established a rat model of EAM, which was the best available model to reflect the DCM process, although it might not completely mimic the physiological condition of human DCM.

For many years, apoptosis was considered the classical cell death pathway to maintain homeostasis in cardiovascular disease. Necroptosis, as a caspase-independent cell death pathway, was considered to be a substituted form of cell death when apoptosis was blocked (33). Recently, several studies indicate that necroptosis and autophagy also occur in various cardiovascular pathological conditions with a faster and more effective cell death response than apoptosis (34, 35). Szobi et al. (5) demonstrated that necroptosis may take place in coronary artery disease (5). Nevertheless, little is known whether three forms of cell death take place together in DCM causing sudden cardiac death.

To understand the mechanism of cell death in DCM in EAM, we first assessed cardiac function and myocardial inflammation by echocardiographic examination and HE staining. Our results showed that cardiac conditions as revealed by EF and FS in the EAM group was significantly decreased on 3W with the presence of dense inflammatory infiltrates in cardiomyocytes, suggesting that EAM was successfully established. Increased necroptosis-mediated cardiac cell death was also observed in the viral cardiomyocyte (VMC) mouse model (7). Next, we further assessed necroptosis-, apoptosis-, and autophagy-inducing genes and protein expressions in EAM. Our Western blotting and RT-QPCR results showed that the expression of RIP1, RIP3, MLKL, and LC3 I and II in the EAM group was significantly increased compared to the control group. mRNA expressions of CASPASE3 and BAX were increased, whereas BCL2 expression was decreased, indicating an increase in apoptosis. Immunohistochemical examination showed that brown particles were all distributed in the myocardial cytoplasm, and we also noticed that the level of necroptosis, apoptosis, and autophagy in the 3W rat group was markedly higher than that in the 8W group. These observations confirmed that all three forms of cell death pathway occurred in the EAM “early” stages (3W) more intensely than in the “late” stages (8W), and thus, we selected the “early” stages (3W) of rats to continue to study the relationship between necroptosis, autophagy, and apoptosis.

To understand the relationship and underlying mechanisms among necroptosis, apoptosis, and autophagy, we treated EAM rats with a necroptosis inhibitor (Nec-1), apoptotic inhibitor (zVAD-fmk), and autophagy inhibitor (3-MA). Nec-1 is a necroptosis-specific small-molecule inhibitor, uniquely inhibiting the activation of RIP1 not through the transcriptional regulation but through the blocking of RIP1 kinase activity and the interaction of RIP1–RIP3 (24, 36). First, we observed that Nec-1 treatment decreased the expression of necroptosis-specific genes and proteins, RIP1, RIP3, and MLKL. Second, the expression of apoptosis-inducing genes CASPASE 3 and BAX was increased but BCL2, a protective gene against apoptosis, was decreased. Thus, blockade of necroptosis by Nec-1 not only prevents necrotic cell death but also induces apoptosis in EAM, although the mechanism of this switching is currently unclear. It is demonstrated that the switch from necroptosis to apoptosis was correlated with the RIP1 kinase activity (4, 37). In cylindromatosis (CYLD), RIP1 recruits CASPASE 8 and Fas-associated protein *via* a death domain (FADD) and TNFR1-associated death domain (TRADD) to trigger apoptosis when CASPASE 8 is activated (37, 38), whereas when CASPASE 8 is blocked, RIP1 and RIP3 interact with each other without degradation and form necrosome to activate necroptosis (25). Our results also indicate that Nec-1 does not have a significant effect on LC3 I and II expression, suggesting that necroptosis inhibition may not affect autophagy. Osborn et al. (39) observed that necroptosis of proliferating FADD-deficient T cells does not depend on hyper-autophagic signaling.

The effect of apoptosis inhibition by zVAD-fmk in EAM induces necroptosis and autophagy. The expression of CASPASE 3 was decreased and RIP1, RIP3, MLKL, and LC3 were markedly increased. Furthermore, in EAM rats, zVAD-fmk improved cardiac conditions, reduced inflammatory infiltration, and attenuated myocardial cell injury compared to the EAM-DMSO group. It is known that apoptosis deficiency can cause a switch from apoptosis to necroptosis, and zVAD-fmk promotes RIP1 phosphorylation by inhibiting CASPASE 8 activation to induce necroptosis (26, 40).

To further determine the relationship between autophagy and necroptosis, we treated EAM rats with 3-MA, an inhibitor of autophagy. Our data showed that the levels of RIP1, RIP3, and MLKL were upregulated in the 3-MA group and thus necroptosis can also be triggered when autophagy is blocked in EAM. We also observed that blockage of autophagy can also improve cardiac conditions and attenuate inflammatory response. Thus, inhibition of autophagy by 3-MA not only switches cell death from autophagy to necroptosis but also protects cardiac conditions from myocarditis. Many studies demonstrated the extensive crosstalk between necroptosis and autophagy as autophagy is capable of inducing, suppressing, or having no effect on necroptosis and apoptosis (39, 41–44). In a cardiac ischemia–reperfusion injury (IRI) mouse model, RIP3K (RIP3 kinase) is shown to decrease mitophagy and promote mitochondria-mediated apoptosis, further implying switching between autophagy and apoptosis (45). In osteosarcoma, autophagy and necroptosis affect the proliferation and invasion of osteosarcoma cells (46). Both cancer and normal cells can induce autophagy and necroptosis in response to nutrient or serum starvation (47). Oberst (48) illustrated that the recruitment of FADD to autophagosomes directly activates RIP1 and RIP3. In contrast, recent studies have also demonstrated that autophagy could inhibit necroptosis under some special conditions, such as stimulation by TNF-α, certain antigen encounters, or starvation (44). We observe that inhibition of necroptosis pathway by Nec-1 may not have much effect on autophagy, whereas inhibition of autophagy increases necroptosis. However, none of the inhibitors reversed LVEF to a normal level. A cell death pathway of necroptosis, or apoptosis, or autophagy cannot be inhibited entirely, but it can activate cells to choose an alternative pathway to participate in cell death (40). These three programmed cell death pathways are not completely isolated, and they are somewhat interrelated. It appears that, in our system, necroptosis and apoptosis are interchangeable and complimentary as blockage of either pathway activates the other. Similar conclusion is suggested by Murphy and Silke (33). In contrast, necroptosis blockage does not have any effect on autophagy, but autophagy blockage increases necroptosis. Thus, taken together, our results suggest that autophagy is an independent cell death pathway that acts upstream of the necroptosis signaling pathway, and it can suppress other types of cell death such as necroptosis. However, it appears that the mechanism of the autophagy pathway is a more complex process.

## Conclusion

**Figure 9:**
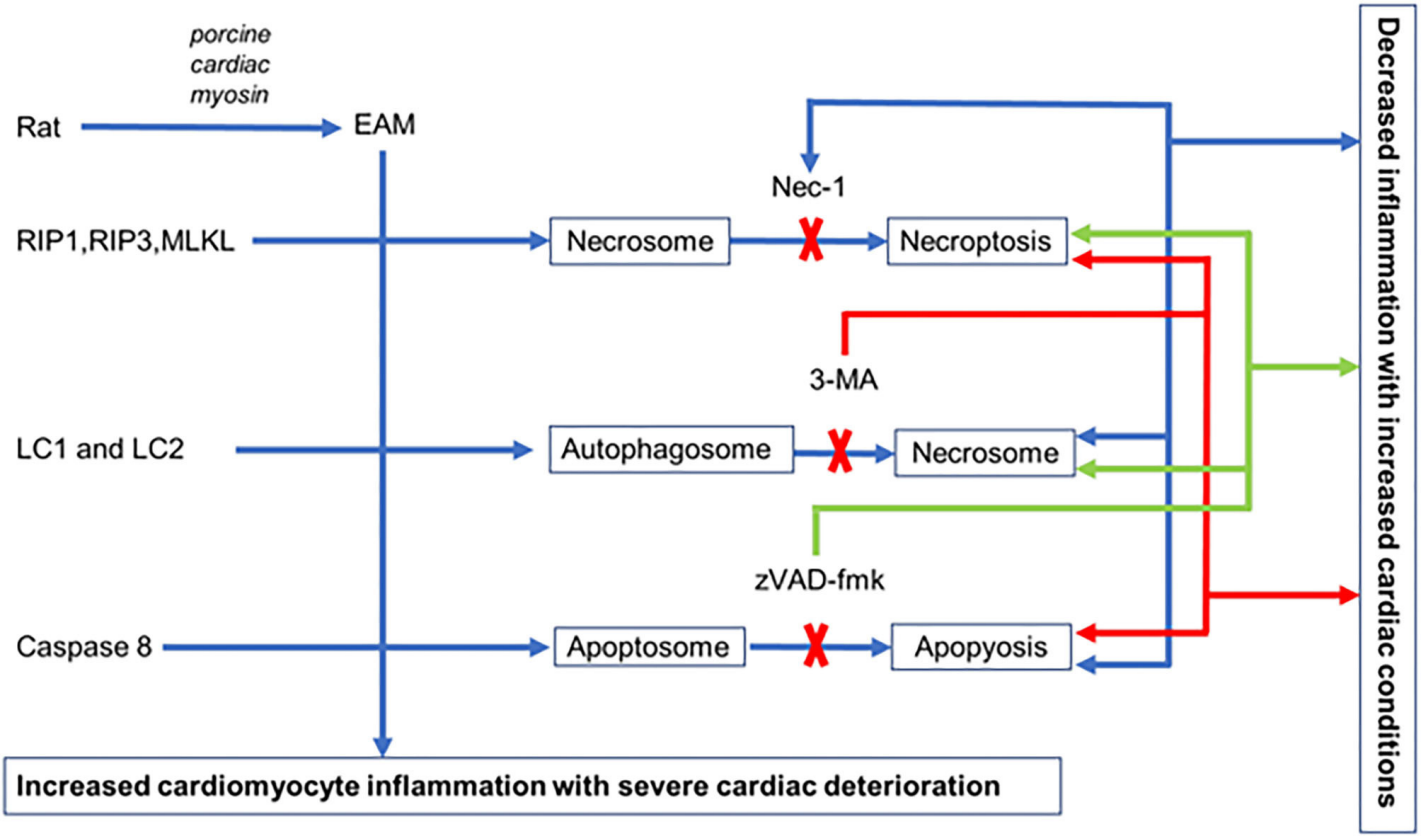
Hypothetical illustration of cell death in EAM model. In EAM, all three processes of cell death take place, but when necroptosis is blocked by Nec-1, apoptosis increases but there is no effect on autophagy. When autophagy is blocked by 3-MA, both apoptosis and necroptosis are increased. When apoptosis is blocked by zVAD-fmk, it increases both necroptosis and autophagy.

We established an EAM model of rats where all three pathways of cell death necroptosis, apoptosis, and autophagy take place with deterioration of cardiac conditions and inflammatory infiltration in cardiomyocytes. Blockage of each cell death pathway, with their respective inhibitor, independently alleviates cardiac conditions with reduced inflammatory infiltration. Each inhibitor not only blocks the specific pathway but also induces alternative pathways to promote cell death in EAM rats. Nevertheless, the interplay between autophagy, apoptosis, and necroptosis is evident in EAM (Figure 9), but the functional role and the interrelated molecular mechanisms of these three cell death pathways remain a subject of further investigations.

## Data Availability Statement

The original contributions presented in the study are included in the article/Supplementary Material, further inquiries can be directed to the corresponding author/s.

## Ethics Statement

The animal study was reviewed and approved by Institutional Review Board of the First Affiliated Hospital of Nanchang University.

## Author Contributions

ZZ and HW: conceptualization and funding acquisition. YW, ZZ, XC, AA, IA, and HW: methodology. YW, ZZ, and XC: validation. YW, ZZ, AM, and HW: formal analysis. ZZ, AM, QY, VN, AA, IA, and HW: writing—review and editing. QY and VN participated in the revision and editing of the paper and the revision of English grammar. All authors contributed to the article and approved the submitted version.

## Conflict of Interest

The authors declare that the research was conducted in the absence of any commercial or financial relationships that could be construed as a potential conflict of interest.

## Publisher's Note

All claims expressed in this article are solely those of the authors and do not necessarily represent those of their affiliated organizations, or those of the publisher, the editors and the reviewers. Any product that may be evaluated in this article, or claim that may be made by its manufacturer, is not guaranteed or endorsed by the publisher.

## Abbreviations

CFA: Complete Freund's adjuvant
CHF: congestive heart failure
CYLD: cylindromatosis
DCM: dilated cardiomyopathy
EAM: experimental autoimmune myocarditis model
EF: ejection fraction
FADD: Fas-associated protein *via* death domain
LVEDd: left ventricular end-diastolic diameter
LVESd: left ventricular end-systolic diameter
3-MA: 3-Methyladenine
MLKL: mixed lineage kinase domain-like
Nec-1: necrostatin-1
PCM: porcine cardiac myosin
RIP: receptor-interacting protein kinase
TNFRT: tumor necrosis factor receptor tumor
VMC: viral myocarditis.
