# Supplementary material for: RIP1/RIP3/MLKL Mediates Myocardial Function Through Necroptosis in Experimental Autoimmune Myocarditis
Source: Front Cardiovasc Med. 2021 Aug 23;8:696362. doi: 10.3389/fcvm.2021.696362 (PMC8419468; doi:10.3389/fcvm.2021.696362)
Supplement: Supplementary file 1 [file Table_1.pdf]

Table 1. The qRT- PCR forward and reverse primers sequences for each gene in rat.

Gene	Type	Sequence	Amplicon
GAPDH	Forward	5`-CGCTAACATCAAATGGGGTG-3`	201
	reverse	5`-TTGCTGACAATCTTGAGGGAG-3`	
RIP1	Forward	5`-CTTGGCACCACCAGATGACTC-3`	161
	reverse	5`-TGTTAGCGAAGACGGCTTGAT-3`	
RIP3	Forward	5`-CTTGAACCCTTCGCTACTGCA-3`	165
	reverse	5`-AAGTAAGCTAGGGTGCCCCC-3`	
MLKL	Forward	5`-AGACTTCTATAAGCCGGGCAAC-3`	181
	reverse	5`-TCACAGCCTTCAAACGGGAT-3`	
Caspase3	Forward	5`-ATGCTTACTCTACCGCACCCG-3`	138
	reverse	5`-GGTTAACACGAGTGAGGATGTGC-3`	
LC3	Forward	5`-CGAGTTGGTCAAGATCATCCG-3`	109
	reverse	5`-GTCAGCGATGGGTGTGGATAC-3`	
BAX	Forward	5`-TGAACTGGACAACAACATGGAG-3`	148
	reverse	5`-AGCAAAGTAGAAAAGGGCAACC-3`	
Bcl2	Forward	5`-TTGTGGCCTTCTTTGAGTTCG-3`	214
	reverse	5`-TTCAGAGACAGCCAGGAGAAATC-3`	
