# Supplementary figures and images for: RIP1/RIP3/MLKL Mediates Myocardial Function Through Necroptosis in Experimental Autoimmune Myocarditis
Source: Front Cardiovasc Med. 2021 Aug 23;8:696362. doi: 10.3389/fcvm.2021.696362 (PMC8419468; doi:10.3389/fcvm.2021.696362)

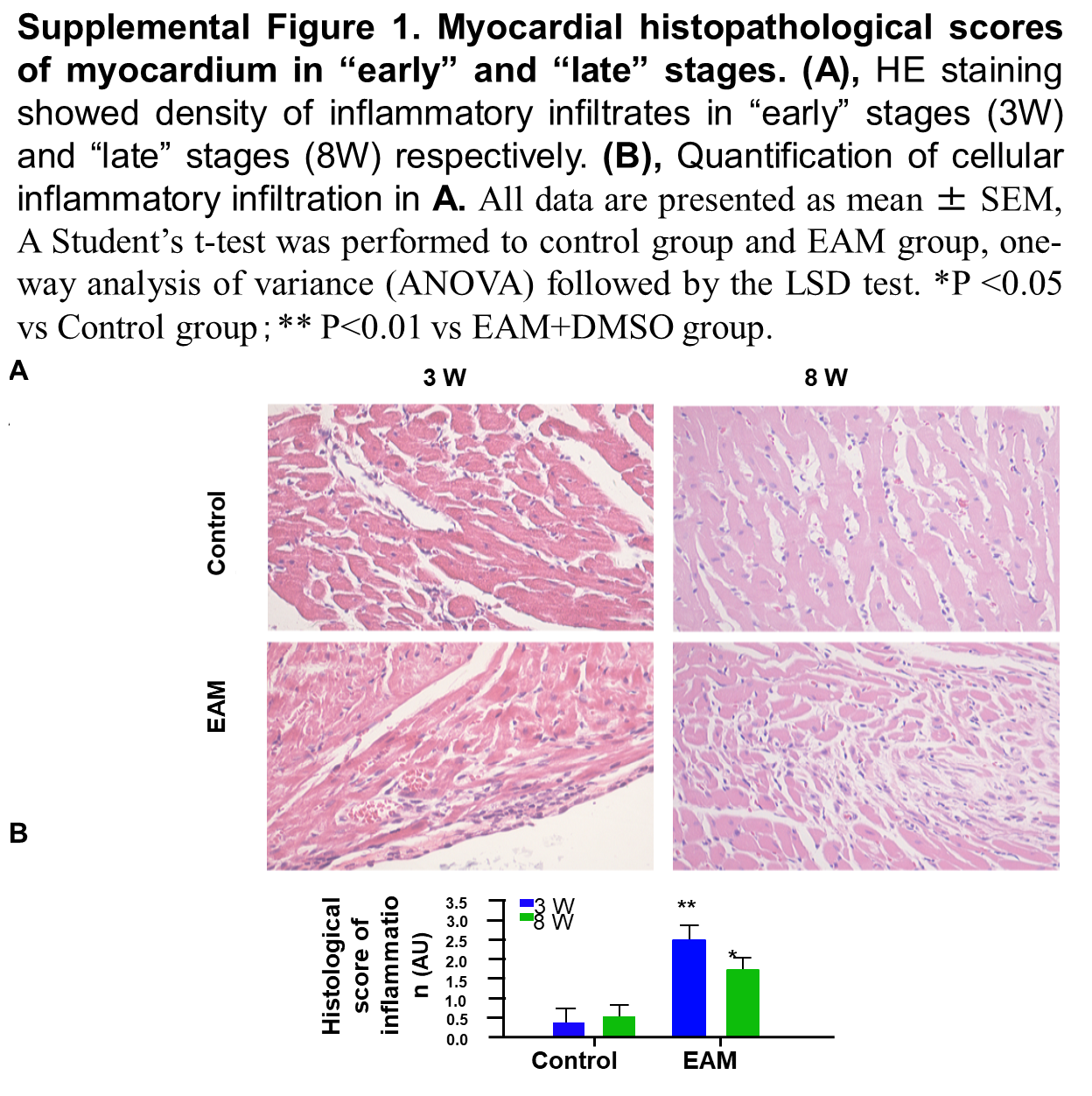

Supplement: Supplementary file 2 [file Image_1.TIFF]

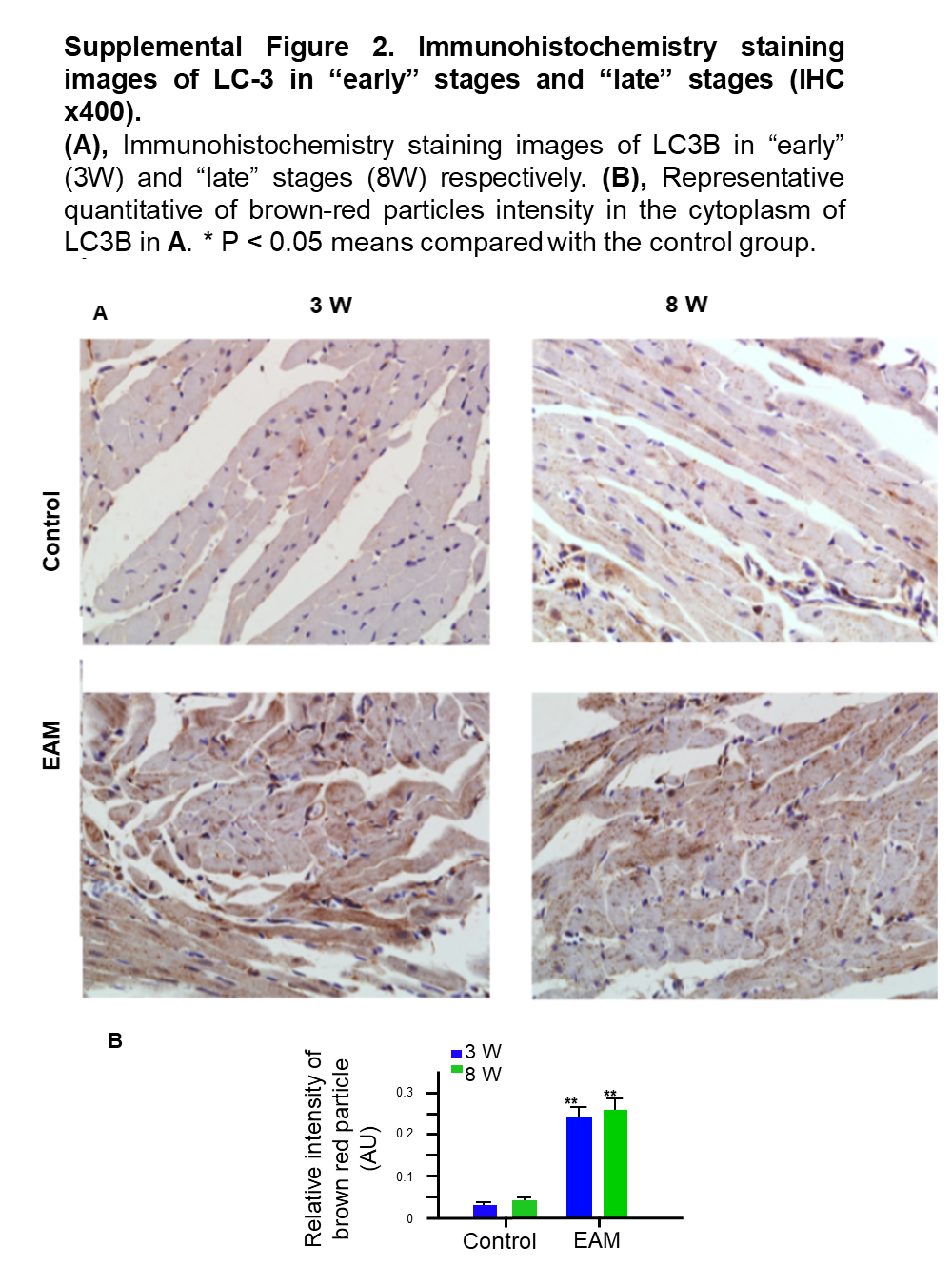

Supplement: Supplementary file 3 [file Image_2.TIFF]

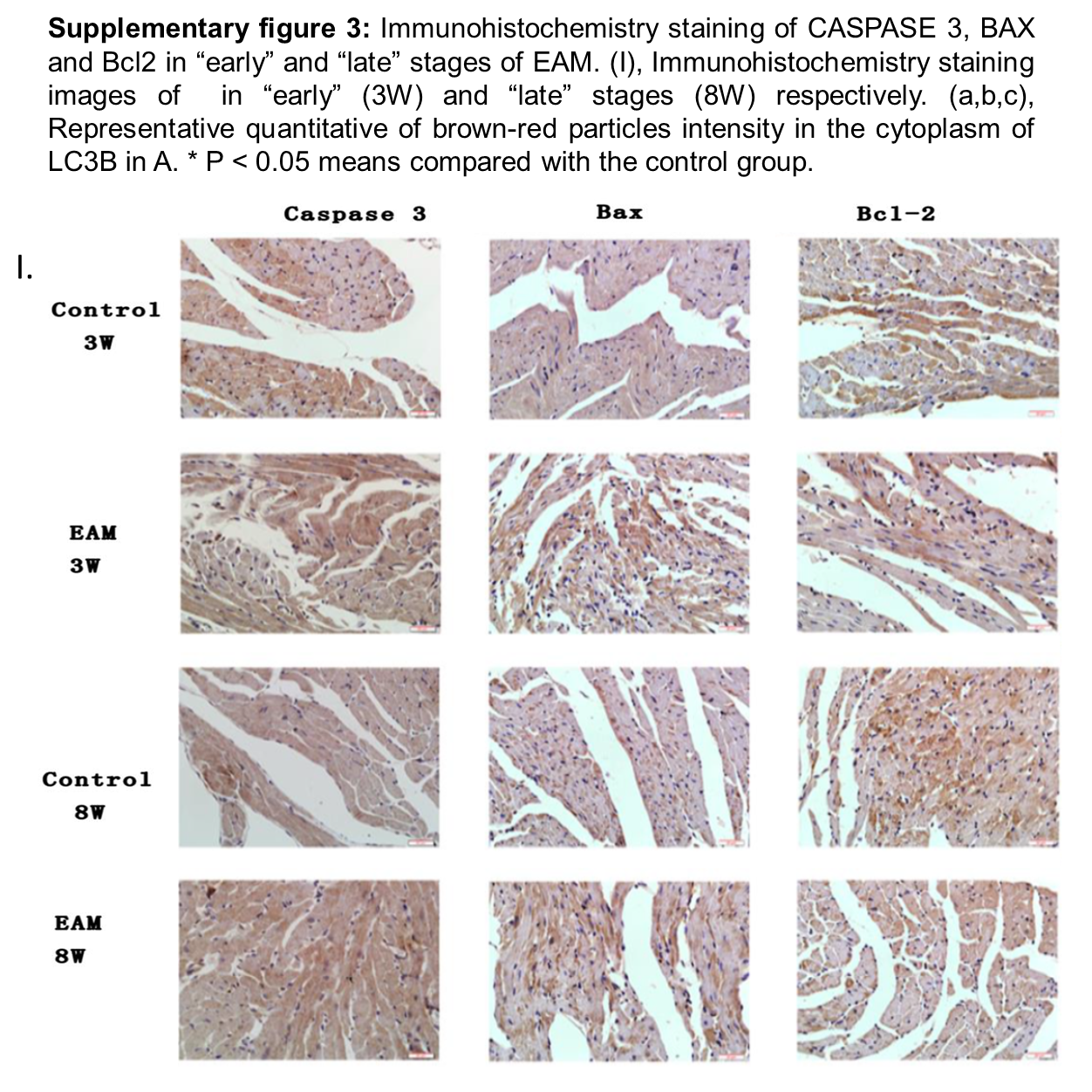

Supplement: Supplementary file 4 [file Image_3.TIFF]
